# Editorial: Immune therapies in neurological disorders

**DOI:** 10.3389/fnins.2025.1615808

**Published:** 2025-05-12

**Authors:** Jie Pan

**Affiliations:** Department of Pathology, Stanford University School of Medicine, Stanford, CA, United States

**Keywords:** immunotherapy, neuroinflammation, CNS, glioma, neurological disorders

## 1 Introduction

Recent years have witnessed an unprecedented surge in the application of immune therapies across a wide spectrum of neurological disorders. From autoimmunity and paraneoplastic syndromes to neurodegeneration and post-injury recovery, immune dysregulation has been increasingly recognized as a key contributor to disease progression. This Research Topic, “*Immune therapies in neurological disorders,”* brings together five diverse yet thematically convergent articles that reflect the translational momentum of immune interventions in neuroscience. These contributions span from mechanistic explorations to clinical applications, offering valuable insights into the evolving immunological landscape of the central nervous system (CNS).

## 2 Bridging oncology and neurology: immunotherapy in paraneoplastic syndromes

The case report by Yang C-H. et al. presents a rare but illuminating case of Anti-Yo antibody-associated paraneoplastic cerebellar degeneration (PCD) secondary to squamous cell lung carcinoma (LUSC). While anti-Yo PCD is predominantly linked to breast and gynecologic cancers (Darnell and Posner, 2003), its occurrence in LUSC highlights the broader implications of onconeural antigen expression and immune cross-reactivity in the CNS. The patient's neurological symptoms improved following combined immunotherapy and cancer treatment, emphasizing the therapeutic window where early immune modulation can rescue neural function. This aligns with emerging data showing that immunotherapies such as IVIG, steroids, or rituximab can modify disease trajectories in autoimmune encephalitis and paraneoplastic disorders (Graus et al., 2016).

## 3 Epigenetics meets immunology: the case of glioma

The bibliometric study by Huo et al. charts two decades of glioma methylation research, revealing the rising prominence of immunotherapy in this domain. Methylation status of MGMT has long been used to predict temozolomide (TMZ) response (Hegi et al., 2005), but newer studies suggest that epigenetic regulation also modulates immune checkpoints, tumor microenvironment (TME), and resistance pathways (Yu and Quail, 2021). Recent efforts have aimed to sensitize gliomas to immune checkpoint inhibitors (ICIs) via combination strategies involving demethylating agents, oncolytic viruses, and local radiotherapy (Hamad et al., 2023). This paper's bibliometric insights reinforce the tight coupling of tumor epigenetics and immunotherapy responsiveness, and serve as a call to integrate epigenetic profiling into future immunotherapeutic trials in gliomas.

## 4 Biomarkers of neuroinflammation: the case of postoperative delirium

Postoperative delirium (POD) is a common neurocognitive complication with significant healthcare implications. The work by Xu M. et al. identifies serum exosomal microRNAs (miRNAs) as potential biomarkers for POD, highlighting miRNA-mediated regulation of neuroinflammatory and synaptic plasticity-related pathways. This adds to the growing body of evidence that systemic immune responses, even in non-CNS surgeries, can impact neurocognitive outcomes via the brain-immune axis. The findings resonate with prior research suggesting that pre-existing neuroinflammation primes microglia for exaggerated responses after systemic insults (Norden and Godbout, 2013). Moving forward, exosomal biomarkers may aid in risk stratification and preoperative screening to mitigate POD risk.

## 5 Stroke and the peripheral–central immune dialogue

In their review, Xu Z. et al. utilize single-cell RNA sequencing (scRNA-seq) to unravel the dynamic immunological interplay in ischemic stroke. Their synthesis underscores the dual role of peripheral immune cells: while acute infiltration contributes to tissue damage, subacute and chronic phases may involve reparative functions. These findings complement recent reports demonstrating that CNS-infiltrating monocytes can either promote neurotoxicity or assist in debris clearance and remyelination, depending on their activation state and local cues (Hammond et al., 2019; Ritzel et al., 2018). scRNA-seq now enables unprecedented resolution in tracking immune cell phenotypes and interactions, and may soon facilitate precision immune modulation in stroke recovery protocols.

## 6 Neurosurgical interventions: opportunities for immunomodulation

The study by Yang S. et al. offers a neurosurgical perspective, comparing two types of titanium mesh in cranioplasty. While this study does not directly investigate immune therapy, it highlights how surgical material and technique can affect recovery and inflammation—an area ripe for exploration. Post-surgical immune responses may not be passive bystanders; they could be modulated to accelerate recovery and minimize long-term deficits (Alam et al., 2018). Furthermore, recent advances have explored coating surgical implants with immunomodulatory agents or incorporating stem-cell-derived exosomes to promote neurorepair (Tomycz et al., 2020; Zhong et al., 2023).

## 7 Beyond the five papers: the expanding horizon of CNS immunotherapy

### 7.1 Checkpoint inhibitors in primary CNS disorders

Immune checkpoint inhibitors (ICIs), such as anti-PD-1/PD-L1 and anti-CTLA-4 antibodies, originally developed for cancer, are now being explored in glioblastoma, primary CNS lymphoma, and even multiple sclerosis (MS). However, their success has been mixed due to the unique immunosuppressive environment of the CNS and challenges in T-cell trafficking across the blood-brain barrier (BBB; Bausart et al., 2022). New approaches—such as intrathecal delivery of ICIs or transient BBB disruption—are under investigation.

### 7.2 CAR-T and engineered immune cells

Chimeric antigen receptor (CAR) T-cell therapy has shown promise in CNS lymphoma, and is being adapted for targeting glioma-specific antigens such as EGFRvIII and IL13Rα2 (Brown et al., 2016). Challenges such as neurotoxicity and antigen escape remain significant but surmountable with next-generation CAR designs, including TRUCKs (T cells redirected for universal cytokine killing) and synNotch-based systems (Roybal et al., 2016).

### 7.3 Microglia and CNS-intrinsic immunity

Recent studies have highlighted that microglia, the resident immune cells of the CNS, exhibit profound functional plasticity. Modulating microglial polarization—between pro-inflammatory (M1-like) and anti-inflammatory (M2-like) states—offers potential for intervention in neurodegeneration, traumatic brain injury, and demyelination (Masuda et al., 2019; Pan et al., 2024a,b). Tools such as CSF1R inhibitors, or even microglia-specific gene editing via CRISPR, are being explored in preclinical models.

### 7.4 Combining immune therapies with gene and cell therapies

The future likely lies in synergy. For instance, immune therapies could be used alongside stem cell transplants to enhance graft integration, or combined with gene therapies to provide localized expression of anti-inflammatory cytokines. The convergence of immunology with bioengineering, systems neuroscience, and artificial intelligence may finally unlock therapeutic windows once considered unreachable in neurology.

## 8 Conclusion: toward a new paradigm

This Research Topic reflects a growing consensus that immune therapies are not merely adjuncts but foundational elements in the future of neurology and neurosurgery. From paraneoplastic syndromes to gliomas, from postoperative delirium to stroke, the centrality of the immune system in shaping CNS pathophysiology and recovery is now undeniable. The challenge ahead lies in translating mechanistic insights into patient-centered therapies—precisely targeted, temporally tuned, and ethically delivered.

We extend our gratitude to the authors who contributed to this Research Topic, and we invite continued cross-disciplinary collaboration to fully realize the transformative potential of *Immune therapies in neurological disorders*.
